# Aging Decreases Hand Volume Expansion with Water Immersion

**DOI:** 10.3389/fphys.2018.00072

**Published:** 2018-02-14

**Authors:** Jamila H. Siamwala, Davina G. Moossazadeh, Timothy R. Macaulay, Rachel L. Becker, Rekha H. Hargens, Alan R. Hargens

**Affiliations:** Department of Orthopedic Surgery, University of California, San Diego, San Diego, CA, United States

**Keywords:** hand volumetry, age, gender, BMI, water immersion, time

## Abstract

Hands may show early signs of aging with altered skin texture, skin permeability and vascular properties. In clinics, a hand volumeter is used to measure swelling of hands due to edema, carpal tunnel syndrome or drug interventions. The hand volume measurements are generally taken without taking age into consideration. We hypothesized that age affects hand volumeter measurements and that the younger age group (≤40 years) records a greater change in hand volume as compared to the older group (>40 years). Four volumetric measurements were taken at 5 min intervals during 20 min of water immersion using a clinically-approved hand volumeter. After 20 min of immersion, the hand volume changes of the younger age group were significantly higher than the older age group (*p* < 0.001). Specifically, the right-hand volume of the younger age group (≤40 years, *n* = 30) increased by 4.3 ± 2%, and the left hand increased by 3.4 ± 2.1%. Conversely, the right-hand volume of the older age group (>40 years, *n* = 10) increased by 2.2 ± 2.0%, and the left hand decreased by 0.6 ± 2.4% after 20 min of water immersion. The data are presented as Mean ± SD. Hand volume changes were not correlated with body mass index (BMI) or gender, and furthermore, neither of these two variables affected the relationship between age and hand volume changes with water immersion. We conclude that the younger age group has a higher increase in hand volume with water immersion as compared to the older age group.

## Introduction

Hand volume measurements by water immersion are used extensively in the clinical setting for measuring swelling of limbs due to post-mastectomy lymphedema (Karges et al., 2003), carpal tunnel syndrome (Braun et al., 1989), rheumatoid arthritis (Rembe, 1970) and post-traumatic conditions (Griffin et al., 1990). The degree of swelling is graded based on the weight of water displaced after hand immersion. Hand volume is typically measured using a hand volumeter, by weighing the water displaced after a limb is immersed in a water-filled hand volume chamber. The volume of the hand is directly proportional to the weight of the water displaced, according to Archimedes' principle. Due to consistent results and ease of performance with limited resources, water displacement is considered a gold standard for hand volume measurements in several diseases. Moreover, this technique is popular in less developed countries with scarce resources and poor clinical facilities. One issue with this technique is that when hand volume measurements are made in the clinic, patient age is generally not taken into account while interpreting the differences in hand volume pre and post-water immersion.

Although the hands of most people are very similar anatomically and physiologically, they are susceptible to the vagaries of aging, as they are one of the most frequently used body parts and exposed to environmental insults on a daily basis. With time and injury, hand functions begin to decline and manifest the effects of aging. Dietary, behavioral, genetic, metabolic and environmental factors are associated with age-related loss of hand dexterity (Flatt, 1960). Dietary factors, including malnutrition, lead to loss of homeostasis of minerals, especially alterations in calcium metabolism. Behavioral factors such as sedentary lifestyle and lack of exercise may also lead to accelerated decline in hand function (Flatt, 1960). Certain genetic factors involving mitochondrial activity, fatty acid metabolism and cancer may influence aging of hands (Reed et al., 1991; Glass et al., 2013). Frequent exposure to ultraviolet light (Akiba et al., 1999) and airborne particulate matter (Kim et al., 2016) may lead to greater aging effects and breakdown of collagen, especially in exposed skin. The skin of the hand is also exposed to other kinds of environmental stresses such as abrasions, lacerations and burns, thus accumulating these insults over a lifetime. Dry skin, which is characteristic of aging, has poor skin barrier functions (Epstein and Maibach, 1965; Plewig, 1970; Rougier et al., 1988), which may affect skin permeability. Thus, the decline in skin permeability with age may be measurable by hand volume changes during water immersion. Anatomical aging of hands affects joints, muscle, tendon, bone, nerve, blood supply, skin, and fingernails. The effects of aging on hand volume after immersion are unknown. In this study, we determined if such age-related effects exist. The hypothesis of the study was that an older age group will have less hand volume change with water immersion as compared to a younger age group.

## Methods

### Participants

Forty participants between the ages of 13 and 65 years participated in this study. Twenty three were males and 18 were females. Thirty participants were in the 13–40 age group, and 10 were in the 41–65 age group. Thirty subjects had a relatively low body mass index (BMI <25), and 10 subjects were overweight (BMI ≥ 25). None of the subjects had cardiovascular problems nor were they taking any medication at the time of this study. The protocol was approved by the Institutional Review Board of University of California, San Diego. Prior to the study, the experimental protocol was explained to the subjects and their written informed consents were obtained. The subjects also signed HIPAA consent forms to grant the researchers access to their medical histories, which they provided as a part of the study. For subjects under 18 years of age, both the subject and the subject's parent or legal guardian signed the consent form.

### Equipment

#### Hand volumeter

All hand volume water displacement measurements were taken using a commercially-available volumeter designed specifically to measure hand volume (Hand Volumeter, Volumeters United, Pheonix, AZ). The device is a clear acrylic box with a stopper in the form of a horizontal bar located at the base to maintain consistency of the hand immersion between subjects. For each experiment, the volumeter was filled with a 3% ethanol 97% water solution to the level of an extended lip through which the displaced liquid flows. The water solution contained ethanol so as to reduce surface tension and improve accuracy and reproducibility of measurements (Hargens et al., 2014).

### Water bath

The subjects' hands were immersed in a large acrylic tub filled with tap water. The water in the tub was kept at constant temperature of 37°C by a heating coil submerged in the water. A thermometer was placed in the water at the beginning and end of each 5 min interval to ensure that the water was maintained at 37°C. Paper outlines in the shapes of typical adult right and left hands were taped on the outside of the acrylic tub on the wall closest to the subject to act as a guide and maintain consistencies in hand position. On the right outline was written “Place right hand here” and on the left outline “Place left hand here” in order to ensure that the subject's hand was in the same position for all four immersion periods of each hand. The subjects were seated for the hand immersion protocol and the chair adjusted according to the height of the individuals. The hands were positioned at the same levels as the heart. This was consistent between the subjects.

### Experimental protocol

Subjects were asked to remove all rings, watches and bands from their hands to eliminate extraneous variables from hand volume measurements. The subjects were also asked to wash their hands with soap solution, rinse and dry them thoroughly before the experiment. The hand volumeter was over-filled with the 3% ethanol solution and maintained at 37°C. A measuring cup was placed under the extended lip of the hand volumeter to collect the excess solution. The filled apparatus was allowed to sit and drain the excess fluid until no more solution flowed out of the extended lip. At this point, a plastic container, which was previously zeroed on a balance (OHAUS Triple Beam Balance 700 Series, accurate to 0.01 g), was placed under the extended lip. The subject's right hand was slowly immersed in the apparatus such that the third web (intermetacarpal) space of the subject's hand placed slight pressure on the stopper rod. The subject's hand was kept in that position until the ethanol solution stopped flowing. At this point, the plastic container containing the displaced ethanol was removed and placed on the weighing balance. The measurement was recorded to 0.01 g after each time the subject's hand was immersed in the hand volumeter. This mass measurement was later converted to a volume measurement (ml) by dividing it by the density of the 3% ethanol solution (0.99367 g/ml). After every measurement, the subject was provided with tissue paper to dry their hand in order to prevent systematic errors.

After an initial baseline volume measurement, the subject's right hand was immersed for 5 min in the 37°C water bath. The subject's hand was relaxed but reproducibly submerged in the water, as guided by the paper outlines. After 5 min, the subject was asked to remove and dry their hand. The steps specified above (measurement of volume displaced and 5 min immersion in 37°C water) were repeated in this order 3 times for a total of 4 rounds and 20 min of immersion in the 37°C water. Following the final immersion in the water, the subject's right hand volume was measured one last time and recorded for a total of 5 measurements of volume displaced. This entire process was then repeated with the subject's left hand in random order.

### Statistics

For statistical evaluation, subjects were divided into two groups: ≤ 40 years and >40 years. The experiments were randomized with either the right hand first or the left hand. The data are expressed as means ± standard deviations of changes in the hand volume with respect to age, time, gender and BMI. The statistical significance between the hand volume changes between age groups (≤40 years and >40 years) was computed using unpaired two-tailed *t*-tests and Mann-Whitney tests. The differences between left and right hand volume changes over time (0, 5, 10, 15, and 20 min), and comparisons by BMI and gender were performed using multiple *t*-tests on the raw values. All statistics were performed in the graphpad prism software. Significance was set at p<0.05.

## Results

### Hand volume changes with age

**Figure 1:**
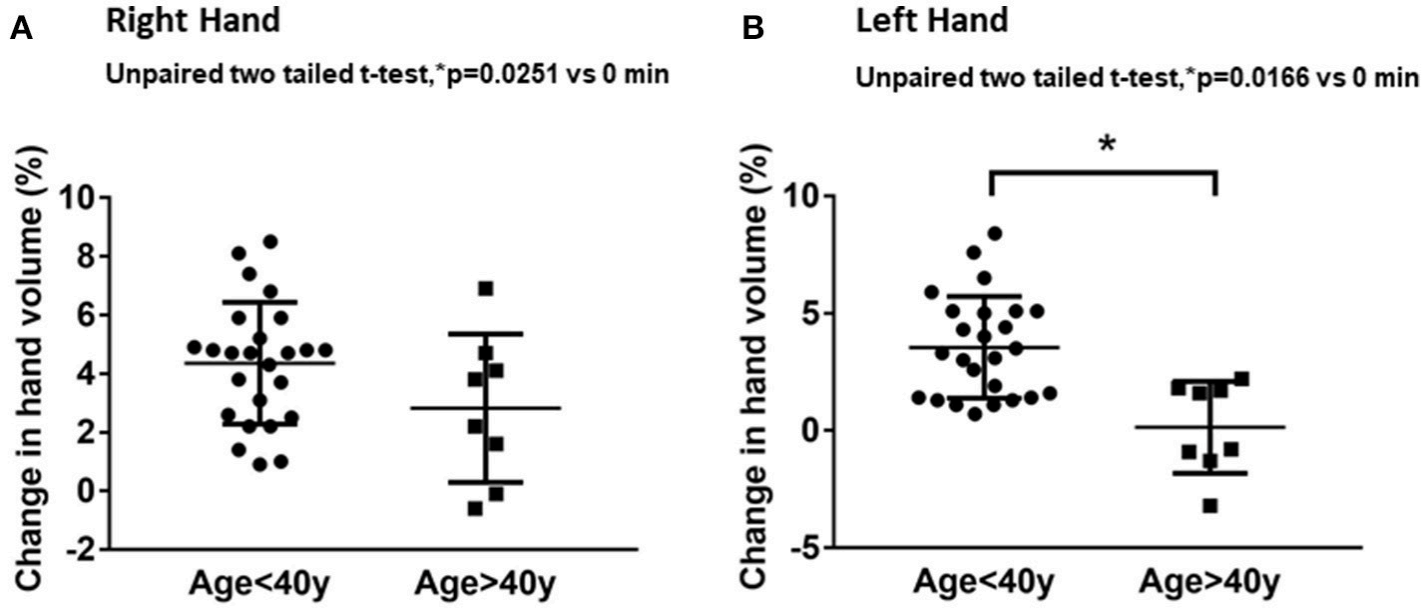
Age-related effects on hand volume measurements. The data represent percentage changes in hand volume measured using a hand volumeter after 20 min of hand immersion in water for the younger age group (≤40 years) and older age group (>40 years) in the right hand **(A)** and left hand **(B)**.Values are means ± SD. ^*^p < 0.05 by two-tailed unpaired *t*-test, *n* = 40.

Volumetric assessments showed significant differences in hand volume measurements between the younger (≤40 years) and older (>40 years) age groups. The younger age group had significantly higher changes in hand volume after 20 min of water immersion compared to the older age group (Figure 1). The right-hand volume change of the younger age group was 4.3 ± 2.0% from baseline compared to 2.2 ± 2.0% in the older age group (Figure 1A). The left-hand volume differences between the younger and older age group were more apparent. The left-hand volume of the younger age group increased 3.5 ± 2.1% from baseline compared to minimal change of 0.6 ± 2.4% from baseline in the older age group (Figure 1B). The hand volume measurements of younger age group were significantly higher than the older age group.

### Association of hand volume changes with age and time

**Figure 2:**
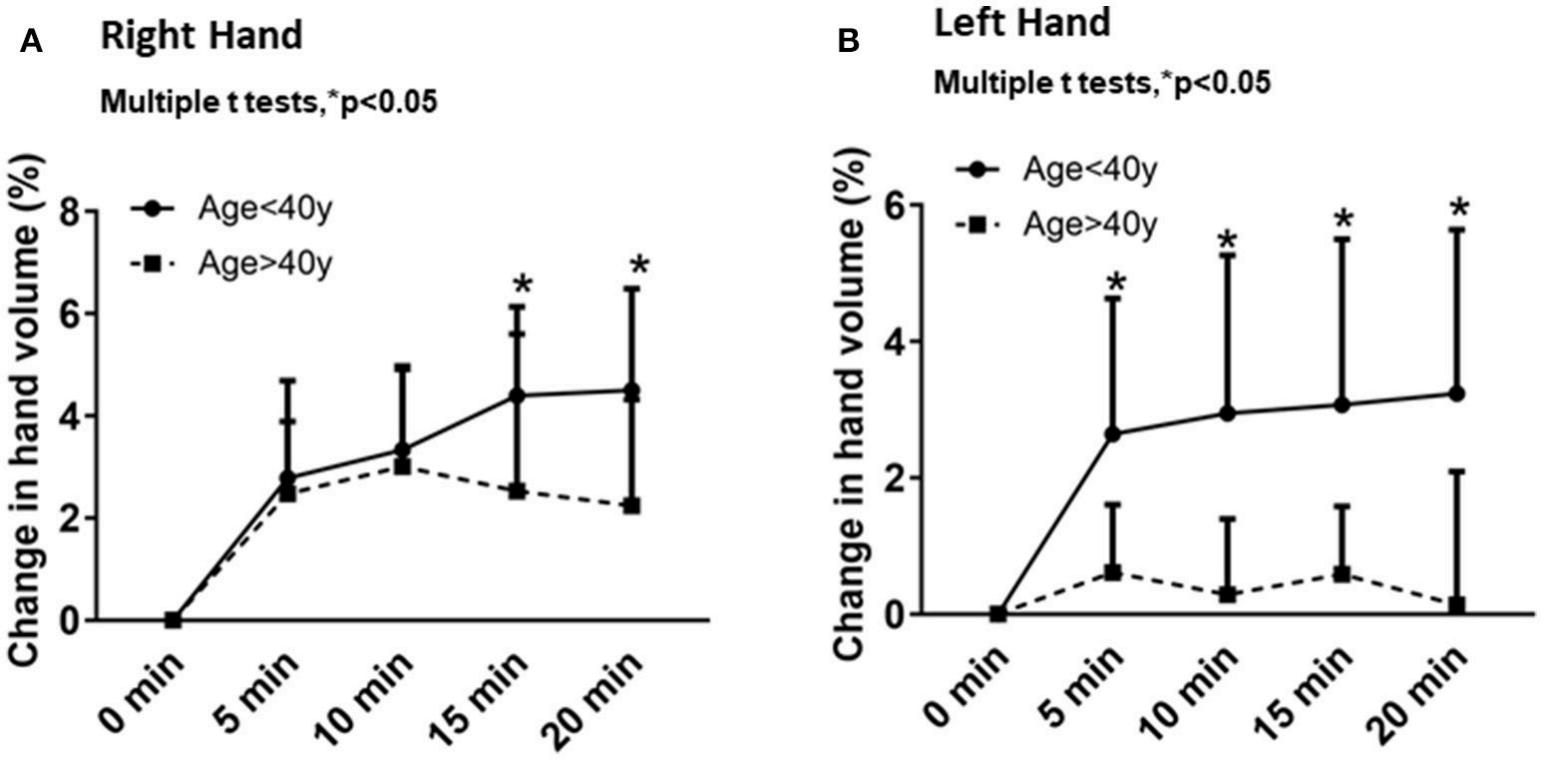
Comparison of the hand volume changes from baseline in each age group for periods of 5, 10, 15, and 20 min for the right hand **(A)** and the left hand **(B)**. Values are means ± SD. ^*^p < 0.05 by multiple *t*-tests.

Temporal changes from baseline in the right and left hand volumes after different times of immersion (5, 10, 15 and 20 min) were plotted for each age group (Figure 2). Overall, the right- and left-hand volumes increased with time (Figures 2A,B). In the younger age group, the right-hand volume changes with time were 2.7 ± 1.9% after 5 min, 3.3 ± 1.6% after 10 min, 4.3 ± 1.7% after 15 min and 4.5 ± 1.9% after 20 min of water immersion. In the older age group, the right-hand volume changes with time were 2.4 ± 1.4% after 5 min, 3.0 ± 1.9% after 10 min, 2.5 ± 3.0% after 15 min and 2.2 ± 2.0% after 20 min of water immersion (Figure 2A). The left-hand volume increased incrementally with time for the younger age group: 2.6 ± 1.9% after 5 min, 2.9 ± 2.3% after 10 min, 3.0 ± 2.4% after 15 min and 3.2 ± 2.4% after 20 min of water immersion. However, the left-hand volume changes decreased with time for the older age group. The left hand volumes were 0.6 ± 0.9% after 5 min, 0.2 ± 1.1% after 10 min, 0.5 ± 0.9% after 15 min and 0.1 ± 1.9% after 20 min of water immersion (Figure 2B). Consistently the older age group showed less change in hand volume over time compared to the younger age group.

### Association of hand volume changes with age and gender

**Figure 3:**
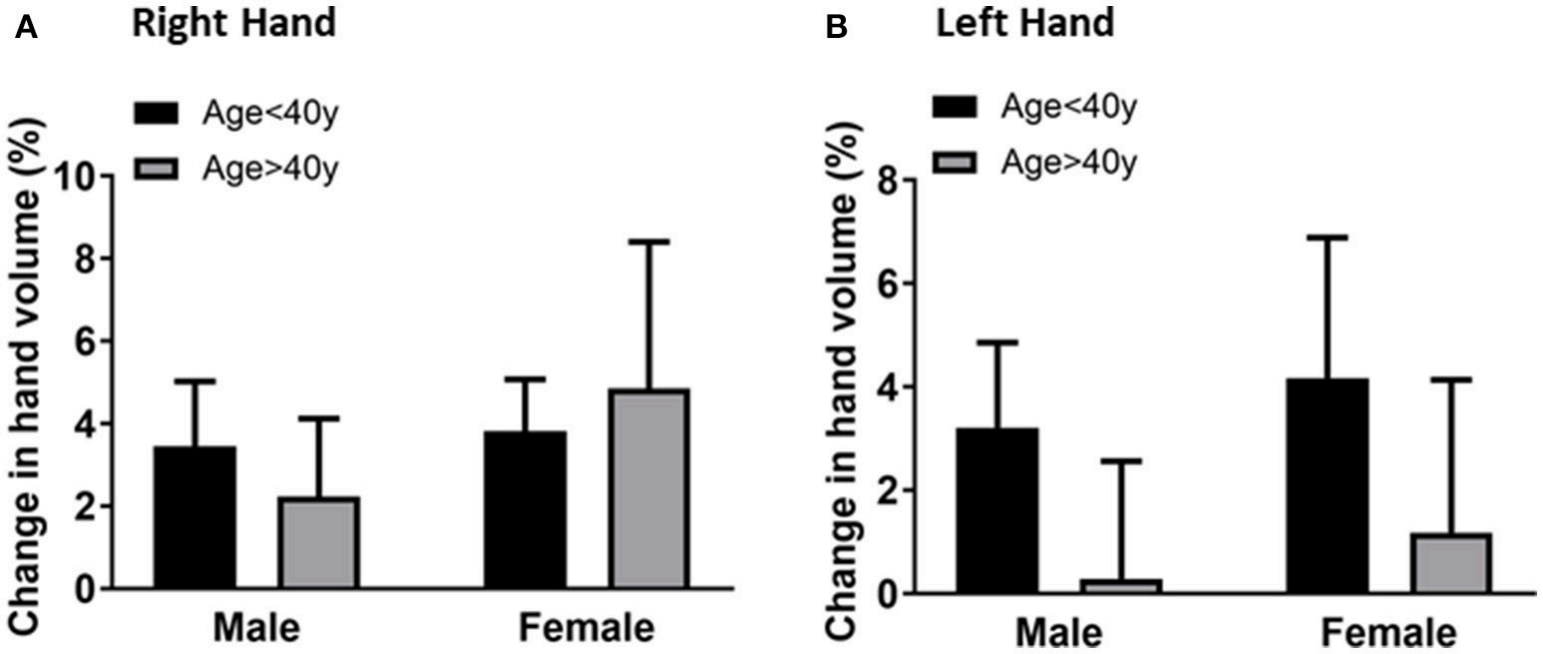
Comparison of the right hand volume changes **(A)** and left hand volume changes **(B)** by gender (male vs. female) after 20 min of hand immersion in water. Values are means ± SD.

To evaluate if hand volume changes were associated with gender, the hand volume changes after 20 min immersion for male and female participants of each age group were compared. The mean for the right-hand volume of males was 3.4 ± 1.5% compared to 3.8 ± 1.2% for females in the younger age group (Figure 3A). The mean for the right-hand volume of older age group males was 2.2 ± 1.8% compared to 4.8 ± 3.5% for females. The mean for the left-hand volume of males was 3.2 ± 1.6% compared to 4.1 ± 2.7% for females in the younger age group (Figure 3B). The mean for the right-hand volume of older age group males was 0.2 ± 2.2% compared to 1.1 ± 2.9% for females. The gender differences are not statistically significant suggesting that gender may not play the primary role in the age-related difference in hand volumeter measurements.

### Association of hand volume changes with age and BMI

**Figure 4:**
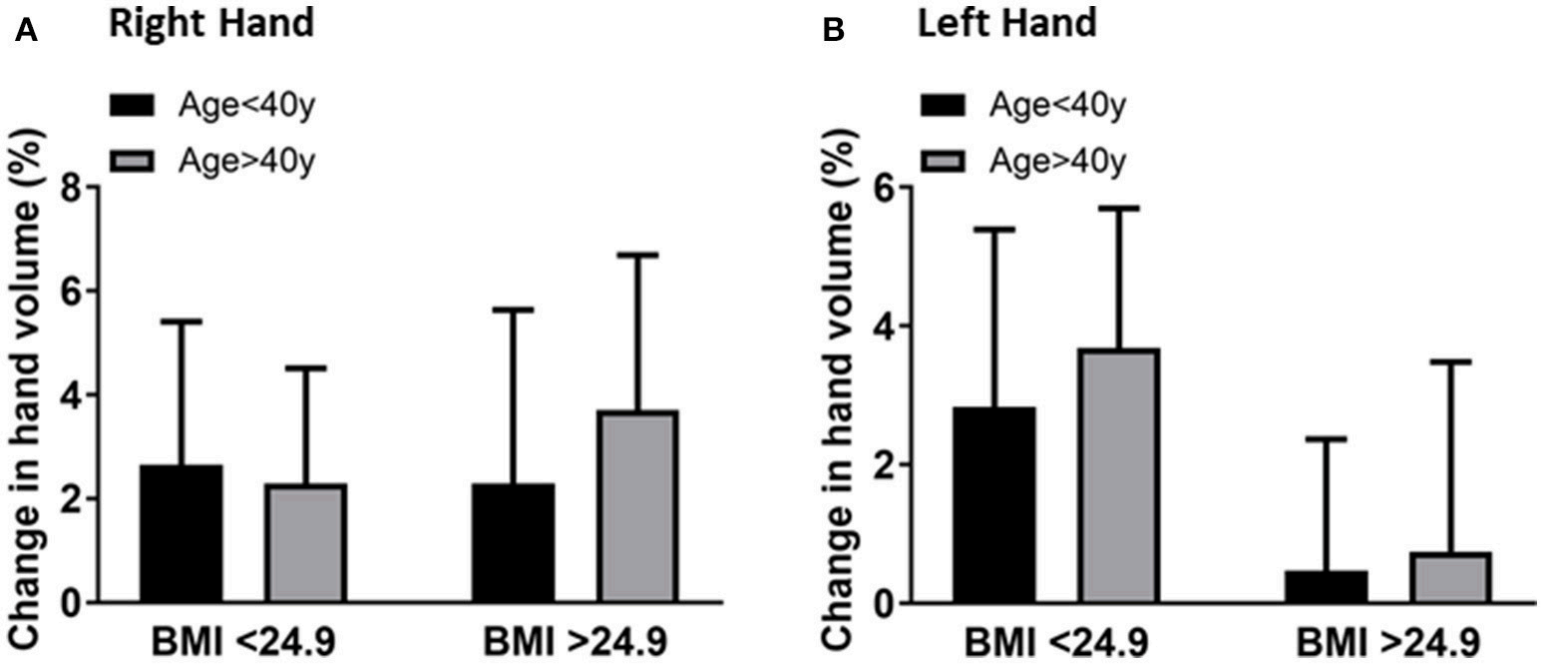
Comparison of the right-hand volume changes **(A)** and left-hand volume changes **(B)** by BMI (<25 vs. ≥25) after 20 min of hand immersion in water. Values are means ± SD. *n* = 40.

The results suggested that BMI may not be associated with age and hand volume changes. Although BMI had no significant effect on the hand volume change, larger hands displace more water compared to smaller hands and the change from baseline was higher in overweight individuals compared to normal weight individuals. However, the effects of BMI on hand volume were independent of age. The right-hand volume change of overweight individuals (BMI ≥ 25) was 2.6 ± 2.2% compared to 2.2 ± 3.7% in normal weight (BMI < 25) individuals in the younger age group (Figure 4A). The left hand volume change of overweight individuals is 2.8 ± 2.5% compared to 0.4 ± 1.8% in normal weight individuals in the younger age group (Figure 4B). This documented that hand volume changes depend more on age and are not associated with the corresponding BMI.

## Discussion

Measurement of hand volume using the hand volumeter is a useful tool in clinics with limited infra-structure to measure edema in disease conditions or after limb swelling surgery. The technique is more sensitive and consistent compared to tape measures used more commonly in the clinics. Our study supports our hypothesis that after 20 min of immersion in water, hand volume of the younger age group (≤40 years) increases more than that of the older age group (>40 years). Gender and BMI have no significant effect on the age-related changes in hand volumes measured using the hand volumeter. Our data have important clinical implications in measurement of hand volume changes with immersion in younger vs. older patients. In other words, patient age must be considered along with disease state.

Certain diseases such as post-mastectomy lymphedema (Karges et al., 2003), carpal tunnel syndrome (Braun et al., 1989), rheumatoid arthritis (Rembe, 1970), and post-traumatic conditions (Griffin et al., 1990) result in the swelling of hands. Apart from tape measurement, a more accurate reading of hand swelling is obtained using a hand volumeter in the clinical setting. The volumeter measurement is considered the most accurate “gold standard” as it can detect small changes in hand volume (DeVore and Hamilton, 1968; Holbrook and Odland, 1974; King, 1993; Hargens et al., 2014). However, age is generally not taken into consideration while measuring swelling of the hand using a hand volumeter.

Our results document that age affects hand volumeter data and therefore should be considered while interpreting the results for patients. Exactly how aging affects changes in hand volume during immersion is not known. Decreased skin permeability and accumulation of dead skin cells with age may be factors that contribute to reduced water uptake during hand immersion with age (Epstein and Maibach, 1965; Plewig, 1970; Rougier et al., 1988).

The skin of the hands mirrors the first signs of human aging, and is reflected by an increase in skin permeability, slow replacement of lipids leading to disturbed barrier function, reduction of cutaneous vascular responsiveness and thinning of the epidermis by 10–50% (Zouboulis and Makrantonaki, 2011). Skin permeability and barrier function are measured by percutaneous absorption and transepidermal water loss through the skin of the hand (Rougier et al., 1988). The size of corneocytes is the main contributing factor for skin permeability. In the elderly, the inward and outward movement of water is correlated with corneocyte size. However, in younger people, corneocyte surface area is low. Thus, percutaneous water absorption and transepidermal water loss increases, but only to a certain point (Rougier et al., 1987). At a surface area of 1,000 μm^2^, there is a limit to the percutaneous absorption or transepidermal water loss. In previous studies of 65 to 80 years olds, the surface area of corneocytes is found to be 20–25% greater than for ages 45–55 years (Plewig, 1970; Rougier et al., 1988).

Skin permeability, changes in corneocyte size and epidermal cell turnover with age may be responsible for the left hand volume changes seen in subjects above the age of 40 years. Apart from corneocytes, the number of sebaceous glands at a particular anatomic site may also influence absorption of water (Blank and Scheuplein, 1969). Another route of water absorption is the transfollicular route in the hands (Scheuplein, 1967). There is an inverse relationship between corneocyte size and epidermal cell turnover. Epidermal cell proliferation decreases with age, which could be another factor responsible for the changes in permeability (Epstein and Maibach, 1965). Specifically, epidermal cell turnover decreases by 30–50% in the third and eighth decade of life (Baker and Blair, 1968; Kligman, 1979). The change in epidermal cell proliferation and keratinization manifests as altered structure and function of the stratum corneum.

Changes in gravitational effects may also affect hand volume changes. Gravity's effects on blood circulation are less in water compared to on land. On land, gravity pulls the blood flow toward the hands and feet in the upright position. However, underwater, the buoyant force of water takes over the functions of gravity and the position of the body is no longer significant. Kraus and colleagues find that by altering local blood pressure by changing the position of the arm, hand volume is affected (Kraus et al., 2013). The question remains if age-related changes in the hands affect hand volume changes with water immersion.

Apart from the changes in skin structure, certain underlying vascular changes also occur with age (Laurent, 2012). Arterial stiffness may affect vascular compliance and decrease absorption of water during hand immersion (Arnett et al., 1994). As early as 40 years, vascular changes may begin to manifest in the form of progressive thickening of the arterial wall layers (intima/media complex), leading to changes in vascular compliance (Arnett et al., 1994). Vascular changes may also include reduction in the number of cutaneous blood vessels, reduced vessel size, increased leakiness of blood vessels and reduced forearm blood flow.

Reduction in the number of microcirculatory vessels and increased arterial stiffness could prevent clearance of transdermally absorbed water from the dermis with aging (Montagna and Carlisle, 1979). Aging is associated with reduction in cutaneous vessel size and loss in vessel density and surface area for exchange. Photoaging, characterized by UV-induced erythema, also has a vascular response similar to that of aged skin. Hence, although differences in hand volume changes are likely related to changes in skin permeability and slow vascular responsiveness, UV-associated aging of the exposed hand should also be taken into consideration. On the other hand, UV exposure also induces upregulation of VEGF and downregulation of angiogenesis inhibitor thrombospondin-1 through MAPK activation in human skin and cultured keratinocytes, leading to leaky vessels and hyperpermeability (Kim et al., 2006). UV exposure third together with chemical aging may be responsible for negative hand volume changes seen by us in the older subjects.

The leakiness of the blood vessels increases with age, and this may contribute to the difference in hand volume change seen in subjects ≤ 40 and >40 years. Vascular endothelial dysfunction is considered as the primary expression of normal human aging. The capacity of endothelial cells to generate nitric oxide (NO) reduces with age (Toda, 2012). Baseline flow mediated, brachial arterial dilatation is also reduced in older subjects compared to younger subjects (Gates et al., 2007). Although both endothelium-dependent and -independent forearm vasodilation are reduced with aging, diseases such as artherosclerosis induce global vascular dysfunction. Reduction in NO with aging may result in vasoconstriction and reduced uptake of water, although we did not measure NO levels in our subjects. Thus, normal and progressive vascular aging may be primarily responsible for the difference in hand volume changes seen in the subjects of different age groups. Hence, all of these factors should be taken into consideration before establishing a standard hand volumeter reading index for different age groups during the routine clinical procedure of hand volume measurements.

### Limitations

Although variables such as water temperature, equipment setup and experiment time were diligently controlled, there are some sources of variability that may limit interpretation of our results. For example, the spill-over time, or drip rate, requires standardization for consistency before measurements. Adding 3% ethanol solution reduces this issue and provides a more consistent measurement of hand volume (Hargens et al., 2014). In addition, the hands of participants must be relatively still to get consistent results. Therefore, this method cannot be used in people with paralysis or Parkinson's disease or diseases in which the hands tremble. Furthermore, skin lesions and open wounds on hands can interfere with the hand volume results.

## Ethics statement

The study was carried out in accordance with the recommendations of the Institutional Review Board (IRB) of the University of California, San Diego and carried out according to the ethical guidelines of the Declaration of Helsinki. Written informed consent was obtained from all participants and their parents prior to study enrollment.

## Author contributions

JS, AH discussed, designed the study and obtained the IRB approvals. RH, JS, and DM performed the experiments. JS, DM, and RH analyzed the results. JS interpreted the results. JS wrote the manuscript. JS prepared the figures. AH revised the manuscript. All the authors read the manuscript for submission.

### Conflict of interest statement

The authors declare that the research was conducted in the absence of any commercial or financial relationships that could be construed as a potential conflict of interest.
